# Monitorization of Varietal Aroma Composition Dynamics during Ripening in Intact *Vitis vinifera* L. Tempranillo Blanco Berries by Hyperspectral Imaging

**DOI:** 10.1021/acs.jafc.2c07425

**Published:** 2023-01-26

**Authors:** Sandra Marín-San Román, Juan Fernández-Novales, Cristina Cebrián-Tarancón, Rosario Sánchez-Gómez, Maria Paz Diago, Teresa Garde-Cerdán

**Affiliations:** † Grupo VIENAP, Instituto de Ciencias de la Vid y del Vino (CSIC, Universidad de La Rioja, Gobierno de La Rioja), Ctra. de Burgos, Km. 6, 26007 Logroño, Spain; ‡ Grupo TELEVITIS, Instituto de Ciencias de la Vid y del Vino (Universidad de La Rioja, CSIC, Gobierno de La Rioja), Ctra. de Burgos, Km. 6, 26007 Logroño, Spain; § Cátedra de Química Agrícola, E.T.S.I. Agrónomos y Montes, Departamento de Ciencia y Tecnología Agroforestal y Genética, Universidad de Castilla-La Mancha, Avda. de España, s/n, 02071 Albacete, Spain

**Keywords:** volatile compounds, partial least squares, total soluble solids, noninvasive, VIS+SW-NIR, TF-SPME

## Abstract

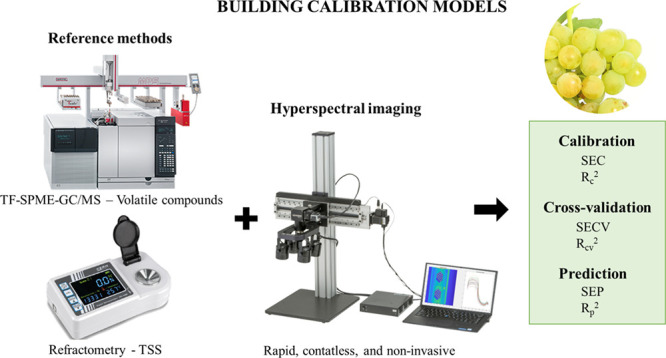

The measurement of aromatic maturity during grape ripening provides very important information for determining the harvest date, particularly in white cultivars. However, there are currently no tools that allow this measurement to be carried out in a noninvasive and rapid way. For this reason, in the present work, we have studied the use of hyperspectral imaging (HSI)) to estimate the aromatic composition of *Vitis vinifera* L. Tempranillo Blanco berries during ripening. A total of 236 spectra in the VIS+short wave near-infrared (VIS+SW-NIR) range (400–1000 nm) of intact berries were acquired contactless under laboratory conditions. As gold standard values, a total of 20 volatile compounds were quantified by gas chromatography–mass spectrometry (GC–MS), and the concentration of total soluble solids (TSS) was measured by refractometry. Calibration, cross-validation, and prediction models were built using partial least squares (PLS). Values of *R*_CV_^2^ ≥ 0.70 were obtained for α-terpineol, *p-*cymene, β-damascenone, β-ionone, benzaldehyde, benzyl alcohol, hexanal, citral, linalool, 2-phenylethanol, octanoic acid, nonanoic acid, 2-hexenal, 2-hexen-1-ol, (Z)-3-hexen-1-ol, total C_13_ norisoprenoids, total C6 compounds, total positive compounds (i.e., the sum of all families except C6 compounds), total benzenoids, and total soluble solids (TSS). Therefore, it can be affirmed that HSI in the VIS + SW-NIR range could be a good tool to estimate the aromatic composition of Tempranillo Blanco grape berries in a contactless, fast, and nondestructive way.

## 1 Introduction

The aromatic compounds that come from the grape, also called varietal aromas, are directly related to the quality of the wine, and therefore to its value.^1−3^ The aromatic characterization of Spanish white grape varieties has been little studied, particularly that of *Vitis vinifera* L. Tempranillo Blanco.^4^ This grapevine variety is a natural mutation of Tempranillo, grown since 2008 only in the Appellation d’Origine Contrôlée (A.O.C.) Rioja, and it is the second white variety (12%) grown in the A.O.C. Rioja in terms of surface area, after Viura (70%).^4−6^

Volatile compounds are found in grapes in concentrations ranging from ng/L to mg/L.^7^ The study of these compounds is very important, as they are directly related to consumer acceptance or rejection^8^ and to the higher or lower trueness to the type of varietal wine within a given viticultural region. Due to the low concentrations at which these compounds are found, sample preparation (extraction, preconcentration, fractionation, and/or isolation) is necessary prior to their chromatographic analysis.^3,9^ These analytical methods are destructive, time-consuming, and require highly qualified personnel and very expensive specific equipment that can only be used in the laboratory and are generally not available in wineries.^10,11^ For these reasons, the evolution of volatile compounds in the berries is not usually assessed throughout the ripening process, which would provide the winemaker with a great deal of information to be able to make decisions regarding vineyard practices (e.g., defoliation around the fruiting zone to increase cluster sun exposure^12^), harvest date, prices according to the grape quality, allocation of the fermentation tank according to the aromatic characteristics of the grapes, etc.^13^ In addition, in recent years, due to climate change, there has been a mismatch between technological maturity (mainly related to the total soluble solids, TSS) and phenolic and aromatic maturities.^14−19^ As a result, berries are often harvested earlier (to avoid high alcohol contents and low acidities in resulting wines), and the adequate content of phenolic and aromatic compounds is not always achieved.^20^ Because of this, rapid and nondestructive methods are being developed that link multivariate spectroscopic and chemical data to predict the concentration of specific chemical components.^20,21^

In the last two decades, hyperspectral imaging (HSI) in the VIS (400–800 nm) and short wave near-infrared (SW-NIR) (800–1700 nm) regions has gained importance as a technology for nondestructive analysis in agricultural applications.^22−25^ While conventional spectroscopy records the response of a small “spot” size, HSI collects the information as a set of images, with each image representing a narrow wavelength range.^22,26^ Therefore, HSI combines two fields, the potential of spectroscopy modeling, with two-dimensional digital imaging, allowing specific regions of either the image and/or the spectrum to be selected, eliminating residual regions. In addition, the acquisition of hyperspectral images can be performed continuously, allowing large areas to be scanned rapidly.^20,23,27^

HSI has been used to estimate the amount, in grapes, of TSS,^10,13,20,23,28−30^ anthocyanins,^10,23,31,32^ amino acids,^13^ tartaric acid, pH, malic acid, total phenols,^10^ total iron-reactive phenolics, tannins,^30,32^ the antioxidant activity, firmness, and hue angle.^30^

There is some work in which HSI has been applied to measure the amount of volatile compounds in single-roasted coffee beans,^33^ in preserved eggs,^34^ and in dry-cured pork.^35,36^ However, to the best of our knowledge, the use of HSI to measure the amount of grape volatile compounds throughout ripening has not been addressed yet. Therefore, the objective of this study was to estimate the grape varietal aromatic composition and TSS of the Tempranillo Blanco variety throughout ripening using HSI, taking data from two vintages.

## 2 Materials and Methods

### 2.1 Materials and Reagents

Chromatographic standards α-terpineol, geraniol, linalool, β-damascenone, β-ionone, benzaldehyde, 2-phenylethanol, benzyl alcohol, octanoic acid, decanoic acid, (E)-2-hexenal, hexanal, 1-hexanol, 2-hexen-1-ol, 2-octanol (internal standard, I.S.), and the reagent NaCl and ethanol (EtOH) were purchased from Merck (Darmstadt, Germany). Water was purified through a Milli-Q system Millipore (Bedfords, MA, USA).

Thin film (TF) with polidimetilsiloxane and carboxen (PDMS/CAR) (carbon fabric film thickness 450 μm), liners packed with Tenax TA, and borosilicate magnetic stirrers were obtained from GERSTEL GmbH & Co (Mülheim an der Ruhr, Deutschland). The BP21 capillary column (50 m length, 0.25 mm i.d., and 0.25 μm film thickness) was purchased from SGE (Ringwood, Australia).

Blender was bought from Philips (Amsterdam, Netherlands). The refractometer, oven, and six-position stirrer plates were purchased from Actylab (Logroño, La Rioja, Spain). Gas chromatography–mass spectrometry (GC–MS) was purchased from Agilent Technologies (Palo Alto, CA, USA). The autosampler system consisted of a multipurpose sampler (MPS) equipped with a tube tray, a thermal desorption unit (TDU), and a cooled injection system (CIS-4) connected to a cryocooling system. MPS and automated TDU were provided from GERSTEL.

### 2.2 Vineyards and Sampling

The clusters of *Vitis vinifera* L. Tempranillo Blanco were hand-picked at random from two rows of a plot belonging to the Gobierno de La Rioja, located in Finca La Grajera (Logroño, La Rioja, Spain) (42°26′26.23″ North Latitude 2°30′51.25″ West Latitude; 447 m above sea level). They were collected, along the ripening period, during two vintages, 2019 and 2020. The vineyard was planted in 2002 following an East–West orientation. At planting, the grapevines were grafted onto 110 Richter rootstock, and they were trained to a vertically shoot-positioned system, with a spacing between rows and within the row of 3.00 m × 1.10 m, respectively, and with a plant density of 3030 plants/ha. During the 2019 vintage, from August to September (from veraison to post-harvest), clusters were harvested on five different dates: 08/12/2019, 08/19/2019, 08/26/2019, 09/02/2019, and 09/09/2019. During the 2020 vintage, from July through September (from veraison to post-harvest), berry samples were collected along seven different dates: 29/07/2020, 05/08/2020, 12/08/2020, 19/08/2020, 26/08/2020, 09/02/2020, and 09/09/2020. At each date, 25 plastic bags were collected with 2–3 clusters of Tempranillo Blanco each. The clusters were frozen at −20 °C until sample preparation.

### 2.3 Sample Preparation

All the clusters picked at a given date were manually destemmed, without defrosting, in a tray. Subsequently, all the berries were mixed in order to achieve the greatest homogeneity. Once homogenized, 64 berries were randomly taken and added to a bag. The process was replicated until 20 samples of 64 berries per date. Sixty-four berries were chosen because it is a multiple of 32, which is the number of berries needed to take the hyperspectral image and also allows to obtain enough must volume to analyze the volatile compounds. At the end of the process, 100 samples were obtained (5 dates × 20 samples/date) in 2019, and 140 samples (7 dates × 20 samples/date) in 2020, making a total of 240 samples pooling together the two vintages.

### 2.4 Calibration Curves

Calibration curves were obtained by the method optimized by Marín-San Román et al.^37^ TF-SPME under 500 rpm stirring, for 6 h, at 20 °C. The standard solutions, which contained different concentrations of each of the compounds, were desorbed and analyzed in the GC–MS, performing three replicates of each one. The solutions were prepared in 50 mL of EtOH. The calibration curve of each compound involves a minimum of four points and a maximum of seven points of different concentrations. The compounds used and the *R*^2^ were as follows: α-terpineol (0.9942), geraniol (0.9154), linalool (0.9643), β-damascenone (0.9684), β-ionone (0.9803), benzaldehyde (0.9684), 2-phenylethanol (0.9853), benzyl alcohol (0.9961), octanoic acid (0.9707), decanoic acid (0.9846), (E)-2-hexenal (0.9937), hexanal (0.9898), 1-hexanol (0.9706), and 2-hexen-1-ol (0.9748). The concentration of the volatile compounds, for which no calibration curve had been built, was calculated using the calibration curve of a compound of the same family, which was in a similar range of concentration. Likewise, for citral, the linalool curve was used; for *p-*cymene, the α-terpineol curve was employed; for acetic, hexanoic, and nonanoic acids, the decanoic acid curve, and for 3-hexen-1-ol, the 2-hexen-1-ol curve was used.

### 2.5 VIS + SW-NIR Hyperspectral Imaging

**Figure 1:**
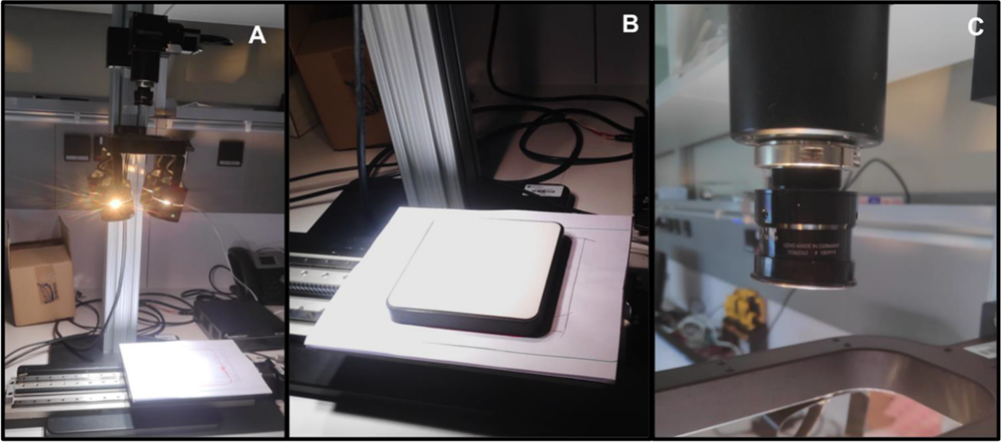
(A) Resonon Pika L VNIR hyperspectral imaging camera, (B) spectralon white reference, and (C) camera lens covered (dark reference acquisition).

**Figure 2:**
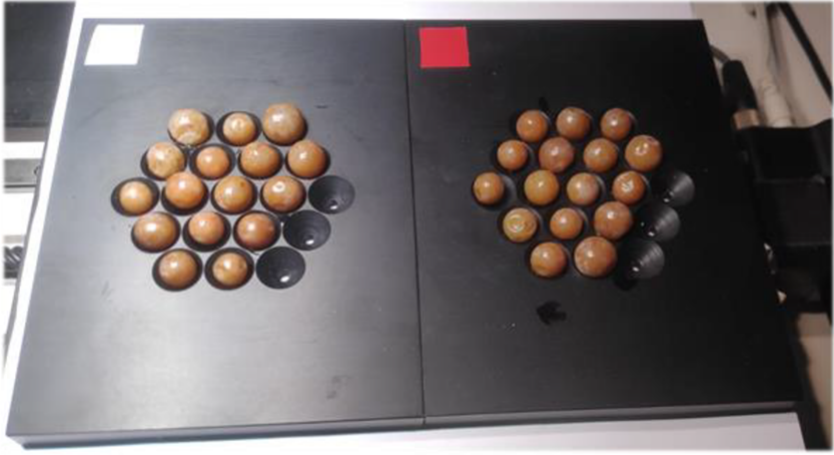
Fruit holders with the first subsample of 32 berries, 16 on each plate, prior HSI.

Hyperspectral images were acquired under laboratory conditions using a push broom Resonon Pika L VIS–NIR hyperspectral imaging camera (Resonon, Bozeman, MA, USA) (Figure 1A). The spectral resolution of the camera was 2.1 nm (300 bands from 400 to 1000 nm), the amount of information captured by the sensor on each spatial line (column) of the hyperspectral image was 900 pixels, and the number of lines was 725. The integration time was set to 30 fps (33.33 ms each line). Therefore, the camera’s time to generate an image was 24.16 s (33.33 ms × 725 lines). The height between the hyperspectral camera and the sample was set to 480 mm and the lighting setup included four 50 w halogen lamps. Prior to HSI, a Spectralon (Labsphere, Sutton, NH, USA) white reference (a surface with a reflectance over 95%) was manually placed at the same distance as the plates (fruit holder) where the berries were placed (Figure 1B). The dark current was measured with the camera lens covered (Figure 1C). After that, for each of the 240 samples, previously defrosted, two hyperspectral images were acquired (two subsamples), with 32 berries for each one (Figure 2). In this way, the 64 berries composing each sample were measured. Berry samples were naturally thawed at ambient temperature and they were carefully dried before measurement with HSI. Between samples, the plate was cleaned with paper to remove previous residues.

The raw camera information, acquired as luminous intensity, was translated into reflectance using eq 1.
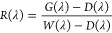
1where λ is the wavelength (nm), *G* is the intensity of the light reflected by the berries (nm), *W* is the intensity of the light coming from the white reference (nm), and *D* is the dark reference (nm). Afterward, the reflectance (R) was converted into absorbance [log (1/*R*)] (nm).

### 2.6 Analysis of Volatile Compounds by TF-SPME and TSS by Refractometry

For the extraction of volatile compounds, the method optimized in Marín-San Román et al.^37^ was used with some modifications. The same 64 berries, from which the hyperspectral image was acquired, were triturated for 30 s in the blender. The paste obtained was introduced into a 50 mL Falcon, and centrifuged at 3900 rpm, for 15 min. An aliquot of 9 mL of centrifuged must sample, 25 μL of the 2-octanol solution (5 μL of 2-octanol/100 mL EtOH), and 2.5 g of NaCl were added in a 10 mL screw-capped vial. A PDMS/CAR TF-SPME device was suspended in the screw-capped vial. A borosilicate magnetic stirrer was added. All samples were stirred at 500 rpm for 6 h at room temperature. After extraction, the TF-SPME device was removed, dried with a tissue paper, and then placed in an empty TDU tube with a glass wool plug at the base. The TDU tube was sealed with a transport adapter and placed in a 98 positions Twister rack on the MPS robotic for automated analysis. The volatile analysis was performed using an automated TDU.

The method used for the determination of the must volatile composition was based on that described by Sánchez-Gómez et al.^38^ with some modifications adapted to the TF-SPME. TF were thermally desorbed in a stream of helium as carrier gas at a flow rate of 75 mL/min in the TDU in splitless desorption mode, increasing the temperature from 40 to 250 °C at a rate of 60 °C/min and holding at the final temperature for 5 min. The analytes were focused on a programmed temperature vaporizing injector (CIS-4), containing a Tenax TA-packed liner with 20 mg of Tenax and held at −40 °C with cryo cooling prior to injection. After desorption and focusing, the CIS-4 temperature was programmed from −40 to 230 °C at 12 °C/s and held at 230 °C for 5 min to transfer volatile compounds onto the analytical column. The CIS-4 operated in solvent vent mode (purge flow to split vent of 80 mL/min at 2 min, vent 60 mL/min, and pressure 20.85 psi).

The desorbed volatile compounds were separated in an Agilent 7890A gas chromatograph system (GC) coupled to a quadrupole Agilent 5975C electron ionization mass spectrometric detector, equipped with a fused silica capillary column (BP21 stationary phase, 30 m length, 0.25 mm I.D., and 0.25 μm film thickness). The helium carrier gas had a constant column pressure of 20.75 psi. The oven temperature of GC was programmed at 40 °C (2 min), raised to 80 °C (5 °C/min, held for 2 min), then to 130 °C (10 °C/min, held for 5 min), then to 150 °C (5 °C/min, held for 5 min), and finally to 230 °C (10 °C/min, held for 5 min). The transfer line temperature was 235 °C. The MS operated in the scan mode (35–180 amu) with ionization energy set at 70 eV. To carry out the identification of each compound, the mass spectra obtained were compared with those of the NIST library and the chromatographic retention index of each standard. Compounds for which no standard was used were identified by comparing their mass spectra with the NIST library. To avoid matrix interferences, it was integrated by extraction ion chromatogram (EIC), isolating the target ion (*m*/*z*) of each compound individually. The target ions (*m*/*z*) were as follows: 41 for 2-hexenal; 43 for acetic acid; 45 for 2-octanol (I.S.); 56 for hexanal, and 1-hexanol; 57 for (E)-2-hexen-1-ol, and 2-ethyl-1-hexanol; 59 for α-terpineol; 60 for hexanoic, octanoic, nonanoic, and decanoic acids; 67 for (Z)-3-hexen-1-ol; 69 for citral, β-damascenone, and geraniol; 71 for linalool; 77 for benzaldehyde; 79 for benzyl alcohol; 91 for 2-phenylethanol; 119 for *p-*cymene; and 177 for β-ionone. Quantification was based on the calibration curves of the respective standards.

The TSS values were measured with the refractometer, adding a few drops of the centrifuged must (15 min, 3900 rpm) and expressed as °Brix.

### 2.7 Spectral Data Analysis

**Figure 3:**
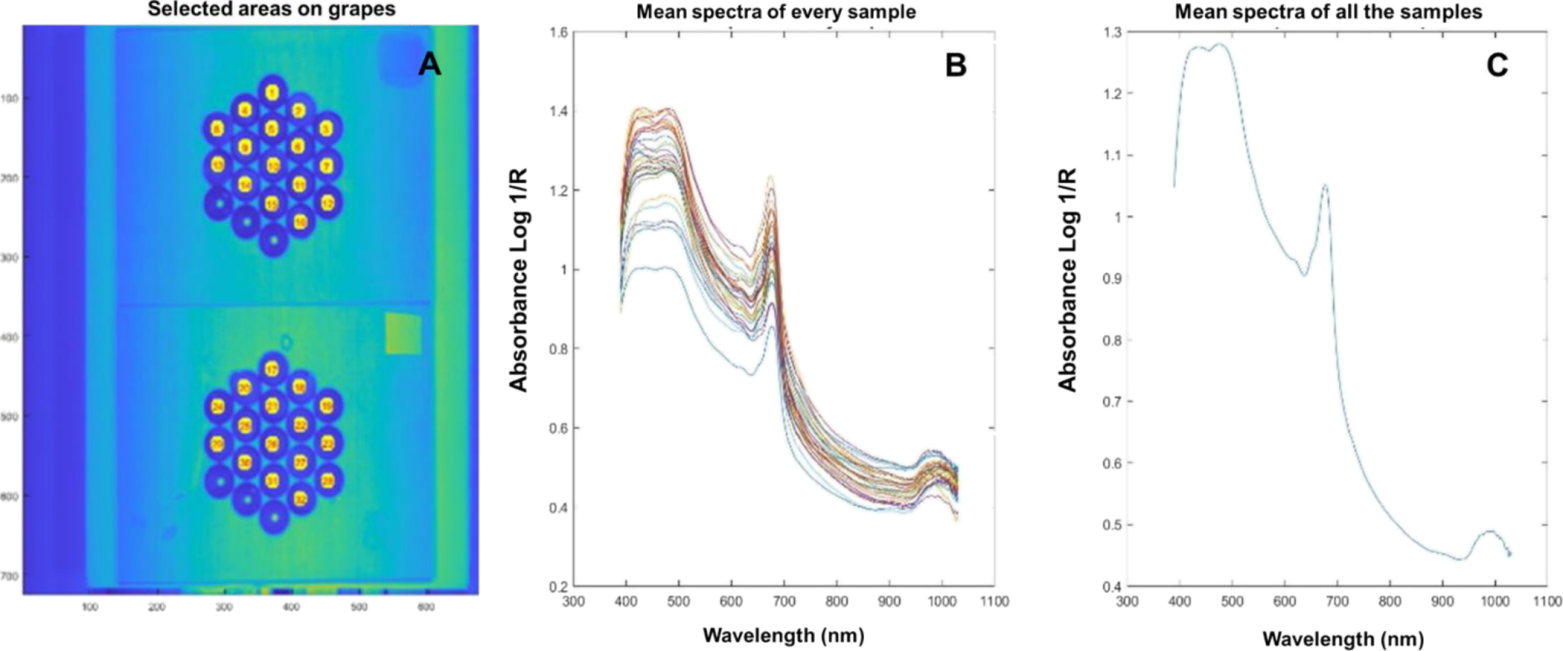
Hyperspectral image from 32 berries of *Vitis vinifera* L. Tempranillo Blanco placed on (A) two fruit holders with the ROIs selected, (B) average absorbance spectra of each berry, and (C) average absorbance spectrum of all berries. R is the reflectance value.

For each hyperspectral image composed of 32 berries placed on two fruit holders, an automated code programmed in Matlab allowed selecting regions of interest (ROI) concerning each one of the 32 berries using a diameter of 7 mm per berry (Figure 3A). The ROIs were extracted and used to calculate the average spectrum of each berry (Figure 3B) and the average of all the berries (Figure 3C) placed on the two fruit holders. Simultaneously, the mean of the pixels comprised in each berry ROI was plotted (Figure 3B) to ensure that the spectral variability remained very similar to the average spectrum of all berries (Figure 3C). Finally, the average spectrum of each sample was composed of two subsamples.

The WinISI II software package version 1.50 (Infrasoft International, PortMatilda, PA, USA) was used for spectral data processing and statistical analysis of hyperspectral images. In a first step, the spectral data were pretreated with standard normal variate (SNV) and detrending to remove the effects of scattering, and to compensate for the baseline offset.^39^ As a second step, the Savitzky–Golay smoothing and derivative process was applied, testing different values for the window size, as well as the first and second derivatives.^40^

In order to explore the data structure, to visualize the presence of outlier spectra, and to identify the main sources of variability in the spectra, a principal component analysis (PCA) was performed with the averaged spectrum of each sample. PCA is an unsupervised pattern recognition technique to provide information about the latent structure of the spectral matrix and to find spectral differences among all spectral samples.^41,42^ Modified partial least squares (MPLS) regression was used for the prediction of the individual and families of the volatile compounds and TSS using the spectra acquired on intact grape berries in the VIS+SW-NIR range 400–1000 nm. The calibration data set was used to train the model, and statistics of calibration and cross-validation, using a fourfold cross-validation approach (to prevent overfitting), were computed to assess the performance of the built models. The validation data set was never used in the training process and was employed for testing with external samples, also called external prediction.

Chemical outliers were eliminated in this process, based on their value in the Student’s *t*-statistic. This statistic indicates the difference between the reference value and the predicted value. A critical limit of *t* > 2.5 was used to identify samples as chemical outliers.^43^ The Studentized residuals from the regression models fitted using least squares is a very common approach to identifying discordant observations in linear regression problems.^44^ In order to train robust models, capable of predicting totally unknown samples, the original data set, with 236 samples (4 samples’ spectra were lost), was divided into two independent data sets: a calibration set, consisting of 80% of the randomly assigned samples (188 samples), and a validation set, consisting of the remaining 20% (48 samples) (Table 1). Each set included samples that were appropriately distributed and covered the entire range of the volatile compound’s concentration and TSS content. Although the same number of samples were used for the data set (236), for the calibration set (188), and for the validation set (48), not all the compounds could be found in all of them, so the N changes for each compound or family (Table 1). Calibration accuracy depends on the standard error of cross-validation (SECV) and standard error of prediction (SEP) used for internal validation or external prediction, respectively. The number of latent variables and determination coefficients of calibration, cross-validation, or external prediction (*R*_C_^2^, *R*_CV_^2^, and *R*_P_^2^, respectively) to represent the proportion of explained variance of the response variables were also computed. The optimal number of latent variables (LVs) was selected as the one yielding the lowest standard error of cross-validation (SECV). Additionally, the residual predictive deviation (RPD), calculated as the ratio between the standard deviation (SD) of the reference data for the training set and the SECV, was also considered (Table 2).

**Table 1:** Descriptive Statistics of 20 Volatile Compounds Content (μg/L), the Total of Each Family Content (μg/L), and Total Soluble Solids (°Brix) of Tempranillo Blanco Grape Berries^a^

compound	data set					calibration set					validation set				
	*N*	minimum	maximum	mean	SD	*N*	minimum	maximum	mean	SD	*N*	minimum	maximum	mean	SD
Terpenoids															
α-terpineol	232	0.022	5.41	0.70	1.15	185	0.022	5.41	0.72	1.18	47	0.024	3.24	0.64	1.03
citral	229	1.24	301.19	34.40	52.84	184	1.24	301.19	36.23	55.72	45	2.80	176.32	26.92	38.50
geraniol	169	0.12	76.85	2.41	6.95	132	0.12	76.85	2.61	7.75	37	0.22	11.90	1.69	2.41
linalool	223	0.33	29.08	3.16	4.53	178	0.33	29.08	3.25	4.67	45	0.44	18.65	2.79	3.93
p-cymene	234	0.032	17.92	0.98	2.33	187	0.032	17.92	1.02	2.47	47	0.038	7.36	0.85	1.70
total terpenoids	234	0.69	333.74	40.09	58.66	187	0.69	333.74	75.15	91.81	47	1.23	204.47	31.27	42.59
C_13_ norisoprenoids															
β-damascenone	230	0.0006	9.63	0.59	1.30	185	0.0006	9.63	0.56	1.27	45	0.0009	6.63	0.70	1.42
β-ionone	234	0.0013	0.59	0.044	0.081	187	0.0013	0.59	0.044	0.082	47	0.0024	0.35	0.044	0.077
total C_13_ norisoprenoids	234	0.0026	10.22	0.62	1.36	187	0.0026	10.22	0.60	1.34	47	0.0035	6.98	0.71	1.47
Benzenoids															
benzaldehyde	231	0.14	10.29	1.69	1.87	185	0.14	10.29	1.71	1.91	46	0.21	6.79	1.59	1.71
2-phenylethanol	234	7.07	330.70	60.90	73.96	187	7.07	330.70	61.76	76.28	47	10.45	318.89	57.47	64.52
benzyl alcohol	233	1.30	90.35	11.79	14.60	186	1.30	90.35	11.75	14.63	47	1.74	77.52	11.95	14.62
total benzenoids	234	9.09	401.78	74.31	89.43	187	9.09	393.11	75.15	91.81	47	12.54	401.78	70.97	80.07
Fatty acids															
acetic acid	234	1.05	221.49	19.09	21.93	187	1.05	221.49	20.03	23.61	47	2.17	48.83	15.32	12.80
hexanoic acid	234	0.59	153.97	10.27	19.84	187	0.59	153.97	11.02	20.97	47	0.77	96.68	7.25	14.26
octanoic acid	234	0.18	17.59	1.87	2.46	187	0.18	17.59	1.91	2.60	47	0.19	7.20	1.73	1.79
nonanoic acid	234	0.053	5.82	0.47	0.61	187	0.053	5.82	0.46	0.62	47	0.11	2.71	0.51	0.57
decanoic acid	172	0.025	3.56	0.15	0.34	137	0.025	3.56	0.15	0.34	35	0.041	1.90	0.18	0.33
total fatty acids	234	1.91	308.92	31.69	38.68	187	1.91	308.92	33.42	41.29	47	4.77	142.15	24.80	24.93
C6 compounds															
2-hexenal	234	19.11	2137.39	274.01	366.20	187	19.11	2137.39	275.28	369.42	47	26.73	1599.21	268.97	356.94
hexanal	234	10.17	3693.21	382.46	590.60	187	10.17	3693.21	373.40	587.16	47	14.56	3344.03	418.57	609.18
1-hexanol	234	18.25	882.20	141.40	108.82	187	18.25	882.20	135.14	102.27	47	28.26	640.62	166.29	130.00
2-hexen-1-ol	182	23.95	7964.66	1032.63	1212.08	144	23.95	7964.66	998.03	1213.56	38	39.75	5029.11	1163.78	1213.52
(z)-3-hexen-1-ol	233	38.77	7680.73	914.07	1098.23	187	38.77	7680.73	899.52	1077.25	46	93.62	6455.88	973.21	1190.42
total c6 compounds	234	344.05	14881.10	2511.20	2392.30	187	344.05	14881.10	2451.86	2363.40	47	543.15	11236.81	2747.27	2516.36
total positive compounds	234	17.53	784.05	146.71	155.02	187	17.53	784.05	151.48	162.25	47	25.29	503.39	127.76	121.77
TSS	236	11.30	24.90	19.47	3.37	188	11.30	24.90	19.41	3.41	48	12.20	24.50	19.67	3.24

a *N*: number of samples in which each compound had been detected; SD: standard deviation; TSS: total soluble solids; total positive compounds: The sum of all families except C6 compounds.

**Table 2:** Calibration, Cross-Validation, and External Prediction Results for the VIS + SW-NIR Models of Volatile Compounds Content (μg/L), the Total of Each Family Content (μg/L), and Total Soluble Solids (°Brix) in Tempranillo Blanco Grape Berries^a^

	spectral treatment	*N*	mean	SD	PLS factor	calibration		cross-validation			external prediction	
compounds						SEC	*R*_C_^2^	SECV	*R*_CV_^2^	RPD	SEP	*R*_P_^2^
Terpenoids												
α-terpineol	Snv-DT + D2W5	147	0.31	0.70	10	0.097	0.98	0.13	0.97	5.38	0.57	0.77
citral	D2W5	143	17.01	20.48	10	5.87	0.92	7.72	0.86	2.65	28.72	0.46
linalool	Snv-DT + D2W5	148	1.97	2.73	10	0.86	0.90	1.08	0.84	2.53	3.57	0.25
*p-*cymene	D1W5	133	0.16	0.24	11	0.062	0.93	0.092	0.90	2.61	1.63	0.25
total terpenoids	D1W5	133	17.51	8.71	8	4.11	0.78	5.01	0.69	1.74	40.23	0.22
C_13_ norisoprenoids												
β-damascenone	D2W5	148	0.12	0.40	11	0.080	0.96	0.11	0.94	3.63	1.20	0.53
β-ionone	D2W5	143	0.018	0.034	11	0.0053	0.98	0.007	0.96	4.85	0.047	0.78
total C_13_ norisoprenoids	D2W5	151	0.17	0.49	11	0.091	0.97	0.12	0.94	4.08	1.16	0.63
Benzenoids												
benzaldehyde	Snv-DT + D2W5	149	1.12	1.25	9	0.27	0.95	0.34	0.93	3.68	1.01	0.66
2-phenylethanol	D2W5	144	29.33	27.78	9	8.22	0.91	10.88	0.87	2.55	62.87	0.16
benzyl alcohol	D2W5	151	7.37	8.94	11	1.98	0.95	2.40	0.93	3.73	12.22	0.34
total benzenoids	Snv-DT + D2W5	153	45.62	53.51	11	13.74	0.93	19.14	0.89	2.79	72.88	0.26
Fatty acids												
hexanoic acid	D2W5	144	3.82	3.17	8	1.47	0.78	1.79	0.68	1.77	14.07	0.16
octanoic acid	Snv-DT + D2W5	142	0.91	0.51	6	0.23	0.80	0.25	0.76	2.04	1.69	0.31
nonanoic acid	Snv-DT + D1W5	149	0.27	0.16	10	0.072	0.80	0.084	0.73	1.90	0.57	0.13
decanoic acid	Snv-DT + D2W5	120	0.079	0.035	3	0.019	0.68	0.021	0.64	1.67	0.32	0.11
total fatty acids	D1W5	151	18.84	11.40	5	8.80	0.40	10.38	0.30	1.10	24.15	0.083
C6 compounds												
2-hexenal	Snv-DT + D2W5	153	146.65	100.28	8	33.77	0.89	40.38	0.84	2.48	313.90	0.58
hexanal	D1W5	163	244.70	170.48	11	44.43	0.93	51.98	0.91	3.27	561.47	0.21
1-hexanol	D2W5	175	119.80	72.47	9	40.20	0.69	46.69	0.58	1.55	116.53	0.29
2-hexen-1-ol	D1W5	122	693.44	697.93	8	237.78	0.89	266.38	0.85	2.62	749.38	0.72
(Z)-3-hexen-1-ol	D2W5	171	723.64	715.37	10	220.45	0.91	270.45	0.86	2.65	748.80	0.66
total C6 compounds	Snv-DT + D2W5	160	1854.12	1511.78	11	385.93	0.93	467.21	0.90	3.23	1804.67	0.60
total positive compounds	Snv-DT + D2W5	162	118.16	127.74	11	28.66	0.95	36.13	0.92	3.54	80.80	0.68
TSS	D1W5	169	19.57	3.44	9	0.38	0.99	0.42	0.98	8.19	1.03	0.91

a Snv-DT: standard normal variate plus detrending; DnWm: Savitzky–Golay filter with “*n*”-degree derivative, window size of “*m*”; *N*: number of samples were the ones used for calibration and cross-validation models after chemical outlier detection (*t* > 2.5); SD: standard deviation; SEC: standard error of calibration; *R*_C_^2^: determination coefficient of calibration; SECV: standard error of cross-validation; *R*_CV_^2^: determination coefficient of cross-validation; RPD: residual predictive deviation; SEP: standard error of prediction; *R*_P_^2^: determination coefficient of prediction. TSS: total soluble solids. Total positive compounds: the sum of all families except C6 compounds.

## 3 Results and Discussion

### 3.1 Volatile Composition of Grape Berries

A total of 20 volatile compounds were identified and quantified in grape berries by GC–MS. Table 1 shows the maximum and minimum, mean, and standard deviation (SD) values for each volatile compound and the TSS in Tempranillo Blanco grape berries. Volatile compounds and TSS were measured from veraison to post-harvest, covering a wide range of concentrations and °Brix, respectively. A total of five terpenoids, two C_13_ norisoprenoids, three benzenoids, five fatty acids, and five C6 compounds were identified. As it can be seen in Table 1, the content of volatile compounds ranged from 0.0006 μg/L for β-damascenone to 7964.66 μg/L for 2-hexen-1-ol.

Terpenoids and C_13_ norisoprenoids are two of the families that contribute most to grape varietal aroma, due to their low perception thresholds.^45^ Terpenoids contribute to the floral and fruity aroma.^46^ C_13_ norisoprenoids, especially β-damascenone, are correlated with fruity aromas. β-Ionone contributes to the violet aroma.^47^

Benzenoids are found in very low concentrations in grapes, like most volatile compounds, but contribute significantly to wine aroma.^48^ As it can be seen in Table 1, 2-phenylethanol is the most abundant benzenoid. This compound is related to the rose aroma descriptor.^49^

It can be observed that the most abundant compounds, in general, were the C6 compounds. This result is consistent with the majority of studies on volatile compounds in grape berries,^50−52^ and particularly in *Vitis vinifera* L. Tempranillo Blanco grape berries as well.^5,53^ C6 compounds are known as “green leaf volatiles” and contribute negatively to wine aroma.^8^ However, since C6 compounds have very high perception thresholds, they have little impact on the final aromatic perception.^47^

Regarding the TSS, the minimum was 11.30 °Brix, which is characteristic of grapes in a phenological stage near veraison, and the maximum was 24.90 °Brix, characteristic of overripe berries of the white variety. It can be observed that the standard deviation (SD) of the compounds was very high, and this is due to the difference between the ripening stages of the fruit. The fact of carrying out such a wide and representative sampling has allowed us to obtain a very robust model. In Table 1, it can be observed that the number of samples (*N*) for each compound was different; this is because not all the compounds were present in all the chromatograms (*N* = 236).

### 3.2 Spectra Analysis

An example of the hyperspectral image from 32 berries of Tempranillo Blanco placed on two fruit holders with the ROIs selected is shown in Figure 3A. In the spectra, three absorption peaks at around 450, 500, and 680 nm can be identified in the visible range. While the first two can be related to the presence of some flavonoids and phenolic compounds bringing the brown-yellowish color to the resulting wines, the peak around 680 is often linked to chlorophyll.^54^ In the NIR region, a wide absorption peak at 976 nm can be clearly observed. This is usually assigned to the second O–H stretch overtone^55^ and related to water absorption. Since grape berries have a high water content, it is often the case that water bands dominate the spectrum in the NIR region.^56^ Must compounds are present in their structure functional groups with O–H, N–H, and C–H bonds, which are responsible for these absorption peaks, so it is feasible that they may relate to the volatile compounds present in berries. Similar spectral properties have been observed in a previous work in which grape mash samples of different grapevine varieties were analyzed for their aroma compounds using NIR spectroscopy.^56^ With regard to the individual aroma compounds and their families, several absorption maxima can be observed in pure signature spectra. However, in complex matrices, such as those from grape berries, they cannot be linked solely to aroma constituents because water, sugars, and acids, which are in much higher concentrations in grape berries, often exhibit the same functional groups.

### 3.3 Chemometric Techniques

Table 2 shows the mathematical pretreatments that provided the best results for calibration (C), cross-validation (CV), and prediction (P) for each of the volatile compounds, as well as for the TSS. As shown in Table 1, the standard deviation (SD) of the volatile compounds was higher than the mean because the grapes were harvested at different ripening times in order to obtain a wide range of both volatile compounds and TSS. Snv-DT means standard normal variate plus detrending. DnWm: Savitzky–Golay filter with an n-degree derivative, window size of m. Volatile compounds whose *R*_CV_^2^ value was greater than 0.30 were included since *R*^2^ values between 0.30 and 0.50 are considered to provide good separation between high and low values. *R*^2^ values between 0.50 and 0.70 provide good separation between high, medium, and low values. *R*^2^ values between 0.70 and 0.90 are considered a good adjustment, and, finally, *R*^2^ values ≥0.90 provide an excellent adjustment.^43^ The *R*^2^ of a set of samples depends mainly on how those samples are distributed within the set. For this reason, the main difference between *R*_CV_^2^ and *R*_P_^2^ is the variability of the data from the calibration set and from the validation set, respectively. As the samples from the validation set were randomly selected and they were far fewer than those from the calibration set, they were not uniformly distributed over the entire range of concentrations, which is why *R*_P_^2^ decreases, and there is such a difference with *R*_CV_^2^.

In Table 2, N means that the number of samples were the ones used for calibration and cross-validation models after chemical outlier detection (eliminating the samples that had *t* > 2.5). The elimination of outliers reduced the range of concentrations covered by the models, leaving out of this range some samples of the validation set (destined for external prediction). This caused a decrease in the prediction accuracy of samples outside this range, increasing the SEP.

The TSS provided a *R*_CV_^2^ ≥ 0.90, as well as the volatile compounds α-terpineol, *p-*cymene, β-damascenone, β-ionone, benzaldehyde, benzyl alcohol, and hexanal, and families of compounds total C_13_ norisoprenoids, total C6 compounds, and total positive compounds (the sum of all families except C6 compounds). These families are determinant in the quality and typicity of the wine because the C_13_ norisoprenoids have very low perception thresholds, so their contribution to aroma is important, and C6 compounds, in high concentrations, contribute negatively to wine aroma.^50,51,57^ For this reason, it is important to find a tool to estimate the concentration of these compounds in grapes before harvesting, to be able to adjust the date of harvest, so the results obtained are very promising. On the other hand, citral, linalool, 2-phenylethanol, octanoic acid, nonanoic acid, 2-hexenal, 2-hexen-1-ol, and (Z)-3-hexen-1-ol, as well as the total benzenoids provided values of *R*_CV_^2^ between 0.70 and 0.90. For all the families and compounds that present a *R*_CV_^2^ ≥ 0.70, a quantitative prediction can be made, which would allow an accurate estimation of the content of these compounds in the grapes. The remaining compounds and families presented values of *R*_CV_^2^ between 0.50 and 0.69, except for the total fatty acids (*R*_CV_^2^ = 0.30). In the work of Caporaso et al.,^33^ in which they estimate the volatile compounds in coffee using HSI, it can also be observed how, in general, the acid models yielded lower *R*_CV_^2^ values than the average of the rest of the volatile compounds. As for the content of volatile compounds, there is only one study that estimates volatile compounds in white grapes (Albariño).^58^ However, in this work, 14 samples of white grapes were used, and the authors did not perform cross-validation or external prediction, so the results obtained in that work cannot be compared with those obtained in the present one. Nevertheless, a very recent work^56^ analyzed grape mash samples from more than 15 grapevine varieties (red and white) grown in Germany using on-line NIR spectroscopy and reported *R*_CV_^2^ values ranging from 0.30 to 0.90 for a series of aroma compounds. Although the aroma profiles reported in the present work and in the German work differ in many compounds, some of them (e.g. linalool, 2-hexenal, 1-hexanol...) can be found in both, and the performance metrics of cross-validation models in these two studies can be compared. Likewise, the *R*_CV_^2^ values for linalool (above 0.84) and 1-hexanol (around 0.58) are very similar in both studies, while the SECV values in the present work were smaller (Table 2) than those in the German study. In terms of the RPD, models for most aroma compounds exhibited RPD values around ∼1.5 in the German work, and between 1.1 and 5.38, with a median value of 2.63 in this study. The RPD indicates the precision behavior of the prediction in comparison with the average composition of all the samples. For this metric, it is usually accepted that models with an RPD smaller than 1.5 are not suitable while those showing RPD values between 1.5 and 2.0 are suitable for differentiating the variability of the data and models while RPDs greater than 2.0 exhibit a very good predictive performance which can be considered excellent when RPDs exceed 3.0.^59^ It can be seen in Table 2 that several compounds have an RPD value greater than 3. Volatile compounds are found in very low concentrations in grapes, so finding a model that allows estimating their concentration throughout ripening, and even quantifying them, was of great difficulty. This fact adds great value to the results obtained in this work.

According to the performance metrics of the present work, the results obtained indicate that there is a possibility of classifying berries according to their high, low, and medium content of these volatile compounds, which could be of considerable benefit to the wine industry. If this equipment is placed in the field, it would be possible to estimate the harvest date based on the aromatic composition of the Tempranillo Blanco grapes, which was not possible until now. On the other hand, if installed in the winery, it would allow classifying the berries into various categories and adding them to different fermentation tanks, looking for different aromatic profiles or wine styles. The values of SECV and SEP were in the range of 0.007–270.45 μg/L and 0.047–749.38 μg/L, respectively, being the minimum SECV and SEP values for β-ionone, the maximum SECV for (Z)-3-hexen-1-ol, and the maximum SEP for 2-hexen-1-ol. The standard error (SE) of a set of samples is proportional to the difference between the reference value and the predicted value and inversely proportional to the total number of samples in the set. For this reason, there is a difference between the SECV and the SEP because the difference between the reference and predicted values is higher in the external prediction than in the cross-validation, and in addition, the number of samples is lower in the external prediction than in the cross-validation, which increases the SEP, with respect to the SECV. The PLS factor ranged from 3 to 11 and the RPD value ranged from 1.10 to 8.19.

**Figure 4:**
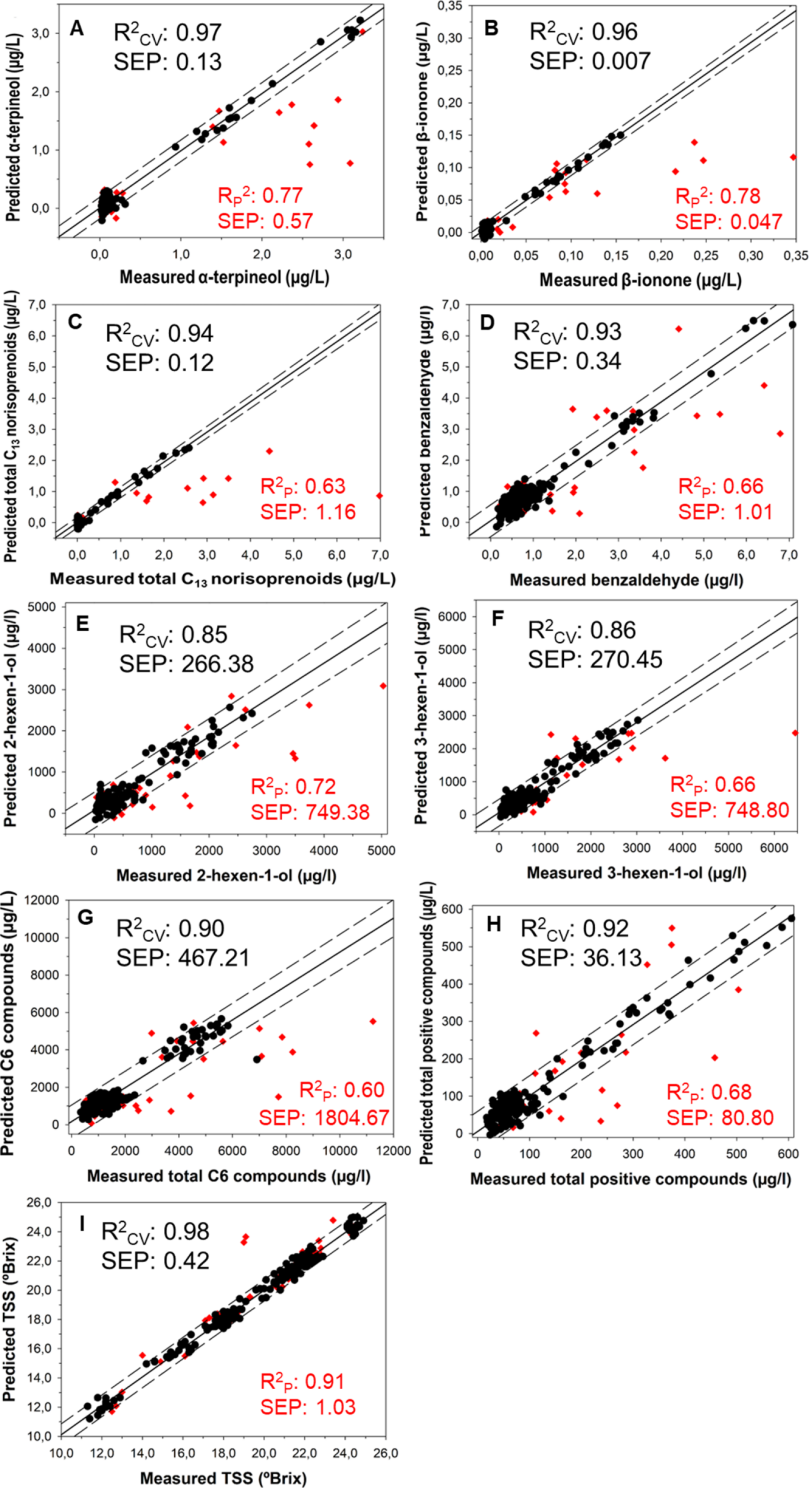
Regression plots for volatile compounds determination using the best PLS prediction models in the VIS + SW-NIR range: (A) α-terpineol; (B) β-ionone; (C) total C_13_ norisoprenoids; (D) benzaldehyde: (E) 2-hexen-1-ol; (F) (Z)-3-hexen-1-ol; (G) total C6 compounds; (H) total positive compounds; and (I) Total Soluble Solid (TSS). Black color: samples of fourfold cross-validation; correlation line: solid line; predictions intervals: dashed lines. Red color: samples of external prediction.

Figure 4 shows the best prediction models for specific volatile compounds in the VIS+SW-NIR: 400–1000 nm spectral range. To facilitate the interpretation of the results (i.e., that is to show a good distribution of data along the regression line), it was decided to plot the chemical families presenting a *R*_P_^2^ ≥ 0.60. The prediction samples (red color) have been plotted together with the calibration model (black color) to facilitate their interpretation. Black samples correspond to the samples used to perform the calibration models (4-fold cross-validation). The number of samples was (Table 2) 147 for α-terpineol, 143 for β-ionone, 151 for total C_13_ norisoprenoids, 149 for benzaldehyde, 122 for 2-hexen-1-ol, 171 for (Z)-3-hexen-1-ol, 160 for total C6 compounds, and 162 for total positive compounds. Red samples correspond to the samples of the validation set, used for external prediction (Table 1).

Considering the large number of samples used to carry out the calibration models (black color), as shown in Figure 4, it can be seen that, for represented volatile compounds, most samples are located in the lowest concentration range (as is normal for volatile compounds in grapes), with a very small number of samples found in higher concentration ranges. This is the reason why the models make the greater error, when making the external prediction (red color), in the samples that are located in the highest concentration ranges. This fact is very well observed in Figure 4A–D where the external prediction samples, with the highest concentrations, fall outside the prediction intervals (dashed lines).

On the other hand, as it can be seen in Table 2, for the TSS, 169 samples were used to perform the cross-validation model (black color). In Figure 4, it can be seen how the samples are more uniformly distributed; this is because the sampling was done at different times of ripening, thus covering a wide range of °Brix. This is the reason why the external prediction comes out so well, with almost all the samples within the prediction intervals (dashed lines). In the case of the TSS, which is the classical and most well-known grape maturity parameter, values of *R*_P_^2^ = 0.91 and SEP = 1.03 were obtained, with RPD well above 3.0 (8.19, Table 2), clearly demonstrating the possibility of accurately estimating this variable using noninvasive HSI. TSS has been studied by HSI in red grapes.^10,23,60−62^ Good results were obtained in all of them, with good correlations and RPD between 1 and 4. On the other hand, there is a study in which HSI is used to estimate the TSS in red grapes and white grapes. In this work they obtained, in white grapes, an RSQ of 0.95, an SECV of 1.10, and an SEP of 1.89.^63^

**Figure 5:**
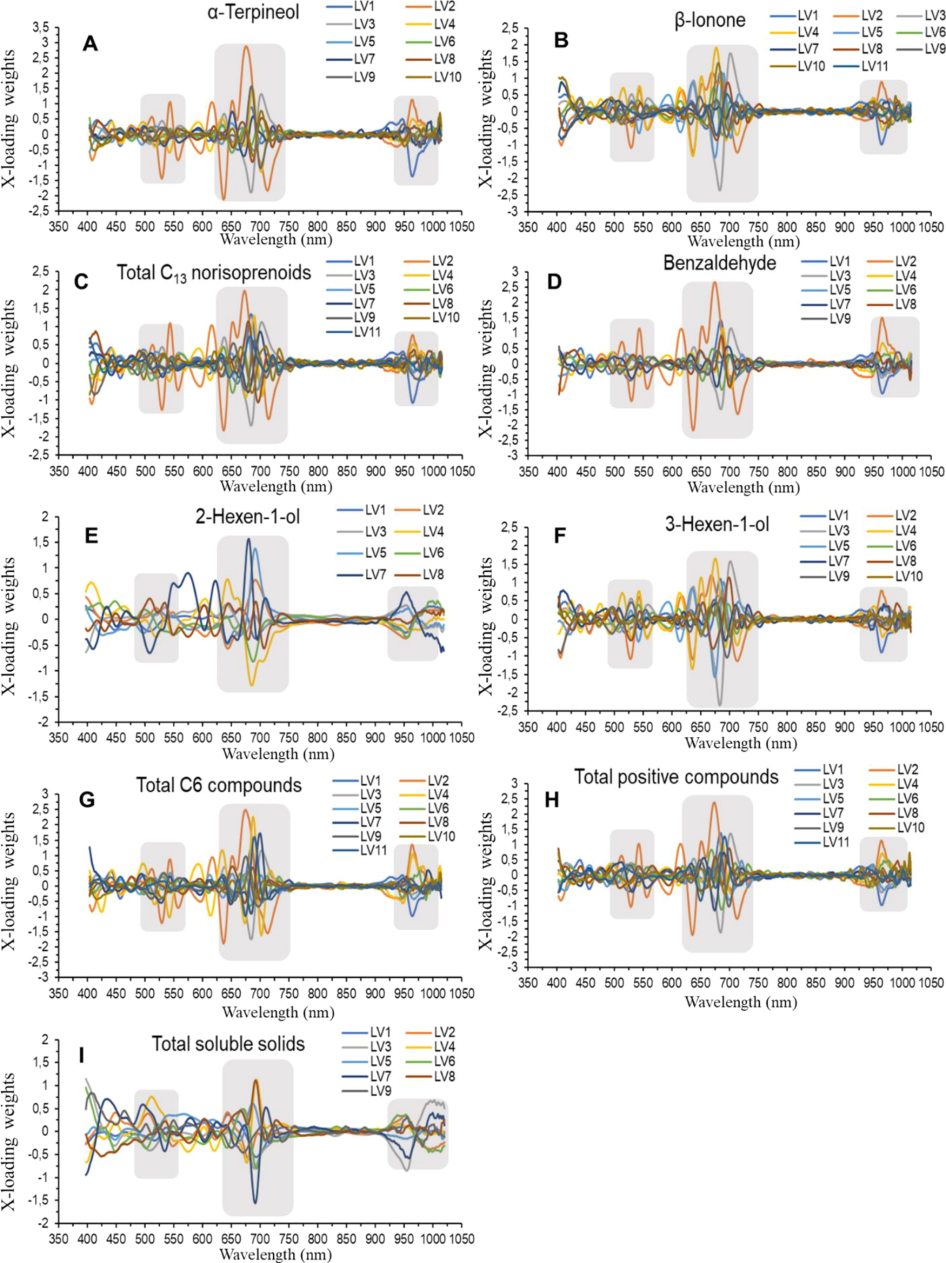
Loading weight plots for all latent variables (LV) of each volatile compounds and TSS determination using the best PLS prediction models in the VIS + SW-NIR range. (A) α-terpineol; (B) β-ionone; (C) total C_13_ norisoprenoids; (D) benzaldehyde; (E) 2-hexen-1-ol; (F) (Z)-3-hexen-1-ol; (G) total C6 compounds; (H) total positive compounds; and (I) TSS. Gray areas indicate the highest loading weights.

Additionally, the loadings for the best PLS prediction models of volatile compounds and TSS, for VIS+SW-NIR: 400–1000 nm spectral range, are plotted in Figure 5. It can be seen how the wavelengths showing the highest weights of the LVs are mainly located in two zones in the visible range, between 500–550 and 600–700 nm, and one zone in the NIR range, between 925 and 1000 nm, the latter usually assigned to the second O-H stretch overtone.

Overall, this novel approach (which is solvent-free, noninvasive, and carried out in intact berries) estimation of grape aroma compounds using HSI can be performed either in-field or at the reception area of wineries for an upgraded characterization of the grape composition and maturity in Tempranillo Blanco. Notwithstanding, it could also be adapted to other cultivars, particularly for the production of super premium wines, in which their quality distinction is not solely made based on TSS or acidity.

In conclusion, a tool has been developed to estimate the aromatic composition and TSS of Tempranillo Blanco grapes in a noninvasive form, using hyperspectral imaging. The results show that the model allows differentiating between high, medium, and low values in all families and compounds, except for total fatty acids. The model also allows quantification of TSS, as well as the content of α-terpineol, *p-*cymene, β-damascenone, β-ionone, benzaldehyde, benzyl alcohol, hexanal, citral, linalool, 2-phenylethanol, octanoic acid, nonanoic acid, 2-hexenal, 2-hexen-1-ol, (Z)-3-hexen-1-ol, total C_13_ norisoprenoids, total C6 compounds, total positive compounds, and total benzenoids in Tempranillo Blanco grapes during the ripening process. The model obtained allows predicting technological maturity and aromatic maturity simultaneously at different stages of grape ripening.
